# Navigating the Lung Transplantation Journey: A Qualitative Study of the Caregivers’ Experiences and Needs

**DOI:** 10.1155/nrp/6853960

**Published:** 2025-12-22

**Authors:** Melissa Gonzalez-Alvarez, María Jesús Megido, Guillermo Pedreira-Robles, Astrid Escrig-Pinol, Roser Escobar-Fornieles, Núria Fabrellas-Padres

**Affiliations:** ^1^ School of Nursing, Hospital del Mar, Affiliated With Pompeu Fabra University, Barcelona, Spain, parcdesalutmar.cat; ^2^ Social Determinants and Health Education Research Group, Hospital del Mar Research Institute, Barcelona, Spain, imim.cat; ^3^ Department of Medicine, Doctorate in Medicine and Translational Research Program, Faculty of Medicine and Health Sciences, University of Barcelona, Barcelona, Spain, ub.edu; ^4^ Primary Health Care Services, Catalan Institute of Health, Barcelona, Spain; ^5^ Jordi Gol i Gurina University Institute for Primary Health Care Research Foundation, Barcelona, Spain; ^6^ Consortium for Biomedical Research in Epidemiology and Public Health, Carlos III Health Institute, Madrid, Spain, isciii.es; ^7^ Department of Respiratory Medicine, Vall d’Hebron University Hospital, Barcelona, Spain, vhebron.net; ^8^ School of Nursing, Faculty of Medicine and Health Sciences, University of Barcelona, Barcelona, Spain, ub.edu

**Keywords:** caregiver, communication, experiences, family, lung transplantation, nursing support

## Abstract

**Introduction:**

The burden of supporting patients undergoing lung transplantation often falls on family members, who become primary caregivers. This role significantly impacts their lives, leading to changes in family roles, physical and emotional exhaustion, and substantial psychological strain. This study explores the experiences, needs, and expectations of family members throughout the lung transplantation process.

**Materials and Methods:**

A qualitative study with a descriptive phenomenological approach was conducted between 2018 and 2022 at Vall d’Hebron University Hospital. In‐depth interviews were performed with 31 adult relatives of lung transplantation patients. Data were analyzed using Colaizzi’s method, identifying central themes and subthemes to better understand participants’ experiences.

**Results:**

Interviewees report having experienced changes in their family and social roles, attending the needs of others more than their own. Although exhausted on many occasions, they are surprised by how they can muster their energy at critical moments. Anxiety and fear are present throughout the process. The future generates uncertainty. The support of their relatives and health staff is crucial for them, but they believe that social and financial support should be increased.

**Conclusion:**

Family caregivers of lung transplant recipients face significant challenges, including role changes, psychological distress, and emotional ambivalence, which align with the transitional experiences described in Meleis’ theory of transitions. Effective support systems, including reliable information, robust social networks, and financial assistance, are crucial for helping caregivers adapt to these demands. Targeted psychological interventions and tailored nursing strategies can mitigate stress, improve caregiver well‐being, and enhance the overall success of the transplantation process. This study highlights the importance of integrating caregiver‐centered approaches into healthcare practices to address the multidimensional needs of this vulnerable population.

## 1. Introduction

Lung transplantation (LT) is a life‐saving surgical option for individuals with advanced lung disease that is refractory to medical treatment [1]. It offers the potential to significantly improve survival and quality of life for individuals with end‐stage lung disease who meet the inclusion criteria at transplant centers. However, LT is associated with unique challenges, including a higher risk of infection and rejection compared to other solid organ transplants [2].

In Spain, LT is performed in eight adult hospitals and two pediatric hospitals. In 2023, 479 LTs were performed (Organización Nacional de Trasplantes [ONT], 2024) [3]. While the geographic distribution of LT centers ensures equitable access for many regions, individuals often need to travel considerable distances to LT centers, which can introduce logistical and financial challenges. Evidence suggests that traveling more than 158 miles is associated with worse survival outcomes after LT, likely due to delayed access to care, difficulties in attending follow‐up visits, and increased caregiver burden [4].

The pathway to an LT is often long and complex, characterized by prolonged illness, reduced autonomy, and diminished quality of life [1, 5, 6]. Recent data from the ONT 2023 report indicate that the average waiting time for LT candidates is 89 days (IQR 30–205 days). The typical patient profile includes a mean age of 57.1 years (SD 10.9), with 65% being men and 35% women [3]. This journey not only impacts individuals but also places significant demands on their families, who often provide essential physical, emotional, and logistical support. Family caregivers play a critical role in supporting patients’ health and quality of life [7], but this role is frequently accompanied by psychological, social, and financial strain [8, 9].

After transplantation, family support remains essential as recipients face limitations in daily activities and require continuous medical follow‐up [8, 9]. Caregivers are often left to navigate these challenges with insufficient professional guidance or social networks [8]. This lack of support exacerbates the physical and psychological burden experienced by caregivers. In a comparative study, Meltzer and Rodrigue [10] found that while liver transplant caregivers reported significantly higher levels of strain than those caring for Alzheimer’s patients, caregivers of lung transplant candidates reported similar levels of psychological burden.

Nurses play a fundamental role throughout the LT process, from the waiting period to postoperative rehabilitation, long‐term follow‐up, and end‐of‐life care. They are uniquely positioned to provide educational resources and emotional support to caregivers, helping them manage stress and adapt to changes in their lives [11–13]. Despite this, the specific needs and expectations of caregivers in the LT context remain underexplored, particularly within the Spanish healthcare system.

While previous studies emphasize the clinical complexity of managing LT patients and the importance of multidisciplinary collaboration to improve outcomes [14, 15], few focus specifically on the lived experiences of family caregivers across the entire transplant trajectory. Existing research often centers on medical or logistical challenges, offers only partial insight into the caregiver role (e.g., in the immediate postoperative phase), or treats caregivers as a secondary source of support rather than as individuals with distinct and evolving needs. To date, no qualitative study in the Spanish context has provided an in‐depth phenomenological analysis of caregivers’ perspectives from the pre‐transplant period through recovery.

Beyond clinical complexity and multidisciplinary coordination, insights from the heart transplantation literature are also informative: caregivers in this context face sustained stress, long‐term caregiving trajectories, and evolving psychosocial needs. A recent qualitative meta‐synthesis in Heart & Lung reports emotionally charged journeys, efforts to balance health and social dynamics, and profound existential reflections among heart transplant recipients, while underscoring the paucity of comprehensive caregiver‐centered evidence and calling for further research on their roles and needs [16]. Taken together, these parallels reinforce the rationale for an in‐depth phenomenological focus on family caregivers across the LT trajectory.

This study addresses that gap in the Spanish context by exploring the experiences, needs, and expectations of first‐degree relatives who support LT recipients throughout the entire process. By highlighting their emotional, social, and informational challenges, the findings aim to inform tailored interventions that enhance the well‐being of both caregivers and patients.

## 2. Methods

### 2.1. Design and Theoretical Framework

This study employs a qualitative approach grounded in Husserl’s descriptive phenomenology [17], which seeks to explore the essence of lived experiences by setting aside researchers’ preconceptions. This framework guided the development of the interview guide and the overall research design, with the aim of capturing participants’ first‐person narratives of caregiving throughout the LT process.

In addition, we incorporated Meleis’ theory of transitions [18] as a complementary theoretical lens to interpret caregivers’ adaptation and role transformation. This theory underscores how personal, contextual, and processual conditions shape individuals’ responses to major life transitions such as caregiving in complex clinical trajectories. Key concepts of the theory include role insufficiency, transition conditions, critical points, and patterns of response—all of which help explain the emotional, relational, and practical challenges faced by caregivers during the transplantation process. Meleis’ framework was used to inform the discussion and to organize the implications of the findings.

For data analysis, we followed Colaizzi’s seven‐step method [19], which is consistent with Husserlian phenomenology, as it emphasizes a structured and rigorous process of distilling meaning units from participants’ descriptions. This method allowed us to identify essential themes that reflect the core structure of the caregiving experience in the context of LT.

#### 2.1.1. Reporting Standards

This study adheres to the Standards for Reporting Qualitative Research (SRQR) guidelines to ensure transparency and rigor in qualitative research reporting [20]. The manuscript follows the 21‐item checklist, including clear descriptions of the research problem, methodological approach, participant selection, data collection, analysis, and ethical considerations, and draws on relevant COREQ items for interview‐based studies.

### 2.2. Setting

This single‐center study was conducted at Vall d’Hebron University Hospital (HUVH), a large public, university, tertiary hospital located in urban Barcelona (Spain). Within Spain’s nationally coordinated transplant system led by the National Transplant Organization (ONT), HUVH serves as the reference program for adult and pediatric LT for residents of the autonomous communities of Catalonia, Aragon, and the Balearic Islands. The study is therefore hospital‐based and embedded in a high‐complexity clinical environment that concentrates pretransplant evaluation, surgery, and specialized follow‐up.

### 2.3. Participants

The participants were 31 first‐degree relatives of adult LT patients whose transplants had been performed between 6 months and 3 years prior to the study. To ensure diverse perspectives, only one family member per transplant patient was selected, except in the case of two daughters who lived together and chose to participate jointly, as both were actively involved in caregiving. Not all participants lived with the transplant recipient, but all met the inclusion criteria of regular involvement in the patient’s care and attendance at outpatient appointments.

The lead researcher had no prior relationship with the participants. Only one participant had previously encountered the researcher while her spouse was in the ICU; however, the researcher did not serve as her primary nurse.

Participants were recruited through a purposeful sampling method, facilitated by the LT team. The initial approach to potential participants was conducted by the case manager, who explained the study and facilitated recruitment. Eligibility criteria required participants to be actively involved in the patient’s care and to regularly attend outpatient appointments. Exclusion criteria included individuals under 18 years of age, those with language barriers, and those not actively engaged in the patient’s post‐transplant care. Some individuals declined to participate in the study.

Active involvement in the patient’s care was operationally defined as providing physical, emotional, or logistical support on a regular basis, and attending follow‐up appointments with the transplant recipient. This definition was applied by the clinical case manager to ensure that all participants had firsthand caregiving experience throughout the transplant process.

### 2.4. Data Collection

Data were collected between 2018 and 2022 through in‐depth, semistructured interviews conducted in person. In line with institutional policy, fieldwork included a temporary pause during the pandemic; COVID‐19 itself was not studied, and references to it are provided only to situate data‐collection conditions.

All interviews took place in a private office within the hospital, ensuring confidentiality and providing a comfortable setting for participants. With their informed consent, interviews were audio‐recorded and transcribed verbatim. Additionally, field notes were taken to capture contextual insights and nonverbal cues, enriching the qualitative analysis.

Recruitment was carried out in two phases. In the first phase, 45 potential participants were approached, of whom 34 agreed to participate. During the second phase, additional individuals were invited to ensure broader representation and thematic depth. Of the 34 who accepted, 31 were ultimately included in the study; three withdrew before their scheduled interview due to changes in personal or caregiving circumstances. No participants were lost to follow up once the interview had taken place.

The interview guide was developed based on the lead researcher’s clinical experience in the intensive care unit, where the emotional burden of family caregivers was frequently observed. This experiential insight was complemented by a narrative review of existing literature on family caregiving in chronic and transplant‐related conditions. The guide was designed to explore physical, emotional, social, and informational aspects of caregiving. Semistructured interviews were selected to ensure thematic consistency while allowing for narrative openness. The final version of the guide was reviewed and refined in collaboration with the thesis supervisors.

All interviews were conducted by the lead researcher, a nurse trained in phenomenology with experience in pulmonary transplantation care, using a semistructured guide reviewed with the thesis supervisors. We did not use moderators or observers; a single interviewer was chosen to reduce procedural variability. The interviewer completed reflexive field notes after each encounter and kept a reflective journal throughout the study to monitor positionality and potential influence.

On average, interviews lasted 40 min. Participants were asked the open‐ended question: “What has your experience of the LT process been like?” Interviews explored physical and emotional health changes, social relationships, financial resources, and expectations for information and support from healthcare and social services. The interview guide was reviewed and approved by the thesis supervisors before data collection began. No formal pilot testing was conducted, but feedback from supervisors ensured its clarity and relevance.

Data collection continued until theoretical saturation was reached, defined as the point at which no new themes emerged during the interviews [21].

#### 2.4.1. Data Collection Timeline and COVID‐19 Context

Interviews were conducted between 2018 and 2022, with a pandemic‐related pause in on‐site research following institutional guidance that suspended nonessential research. Recruitment and interviewing resumed progressively as clinical operations stabilized and a controlled normality returned. Prior to the national lockdown, the LT unit already promoted strict infection prevention practices, so pandemic changes largely intensified existing precautions (e.g., selective telemedicine for follow‐up and avoidance of nonessential hospital visits).

Importantly, COVID‐19 was not an object of inquiry in this study; references to the pandemic only contextualize data‐collection conditions. During analysis, we noted interview timing (pre‐COVID/COVID) and used reflexive memos to examine potential temporal nuances. The core thematic structure remained stable across periods. Notably, fear and hyper‐vigilance regarding rejection and infections were salient both before and during the pandemic; COVID‐19 primarily influenced care logistics and presence at appointments/visits, rather than altering the underlying experiential themes.

### 2.5. Data Analysis

We conducted a thematic analysis following Colaizzi’s seven‐phase process [19], which includes: (1) reading all participants’ descriptions; (2) extracting significant statements; (3) formulating meanings; (4) organizing meanings into theme clusters; (5) developing an exhaustive description; (6) identifying the fundamental structure; and (7) validating findings through participant feedback. All interviews were transcribed verbatim and imported into ATLAS.ti software for systematic coding and the organization of data into categories and subcategories.

Analysis was iterative and phenomenologically oriented, involving multiple readings and constant comparison within and across cases and phases of the LT trajectory. Subcategories were first developed to capture specific facets of the experience and were then consolidated into overarching themes.

To ensure analytic rigor, we combined investigator triangulation and participant validation. Two researchers from the broader team—not involved in initial coding—independently reviewed selected coded transcripts and the evolving thematic structure; this external scrutiny supported consistency and helped minimize individual bias.

Participant validation was conducted ethically and voluntarily. At the end of each interview, the researcher explained the possibility of later contact and invited participants to indicate their preferred method of communication. While most provided a phone number, many declined to participate in the second phase. Four participants reviewed the thematic categories via email. To reduce burden, full transcripts were not shared; instead, participants received a summary of the main categories with illustrative excerpts. Their feedback was recorded in the reflective journal and incorporated into the final interpretive framework [22].

We also undertook peer debriefings at key junctures: the lead researcher met regularly with the thesis supervisors and coauthors (including a qualitative‐methods expert and a clinician‐researcher in solid‐organ transplantation) to review the codebook and thematic map, challenge early interpretations, and examine discrepant cases. An audit trail (versions of codebooks, decision logs, analytic memos) and the interviewer’s reflective journal documented analytic decisions and positionality, supporting credibility, dependability, and confirmability.

Discrepancies in theme identification or interpretation were resolved through iterative discussion within the team, refining code definitions and category boundaries until consensus was reached. Reporting adheres to SRQR and draws on relevant COREQ‐informed items for interview‐based studies to maximize transparency.

### 2.6. Ethical Considerations

The study was approved by the Clinical Research Ethics Committee at HUVH (PR[AG]11/2018). Participants received detailed verbal and written information about the study and provided informed consent before participation.

## 3. Findings

This study explored the experiences and needs of 31 first‐degree adult relatives and partners of 30 LT patients, providing valuable insights into their roles and challenges during the post‐transplantation journey.

Table 1 provides an overview of the participants’ sociodemographic characteristics. The mean age was 52 years, and 77.4% identified as women. Most participants were the patients’ partners (*n* = 21), followed by daughters (*n* = 5), sisters (*n* = 4), and one mother (*n* = 1). Although occupational data are not included in the table for clarity, participants came from diverse professional backgrounds. The distance between their homes and the hospital ranged from 2 km to 622 km, illustrating the variability in geographical and contextual circumstances that shaped their caregiving experiences. Only the most relevant variables are presented to enhance readability.

**Table 1:** Participant and interview characteristics.

Participant	Age	Gender	Kinship	Months since LT	kms between home and hospital
P1	50–60	Female	Wife	11	2
P2	40–50	Female	Wife	13	307
P3	50–60	Female	Sister	10	31
P4	60–70	Male	Husband	14	38
P5	40–50	Female	Wife	6	109
P6	30–40	Female	Daughter	9	22
P7	20–30	Female	Daughter	9	22
P8	50–60	Male	Husband	28	64
P9	60–70	Female	Sister	27	12
P10	60–70	Female	Wife	26	25
P11	50–60	Female	Wife	9	110
P12	60–70	Male	Husband	9	12
P13	50–60	Female	Wife	24	5
P14	50–60	Female	Wife	24	5
P15	60–70	Male	Brother	12	5
P16	50–60	Male	Husband	13	7
P17	40–50	Female	Sister	8	5
P18	60–70	Female	Wife	18	10
P19	20–30	Female	Daughter	16	109
P20	40–50	Female	Wife	9	207
P21	40–50	Female	Daughter	28	32
P22	50–60	Male	Husband	10	110
P23	40–50	Male	Husband	6	622
P24	50–60	Female	Wife	14	16
P25	20–30	Female	Daughter	28	155
P26	50–60	Female	Wife	33	6
P27	60–70	Female	Wife	30	10
P28	50–60	Female	Wife	10	12
P29	60–70	Female	Wife	11	22
P30	70–80	Female	Wife	6	75
P31	50–60	Female	Mother	7	42

Thematic analysis ultimately identified four main categories, each containing several subcategories, providing a nuanced understanding of the multifaceted roles, challenges, and expectations faced by family caregivers throughout the LT journey. These categories and their subcategories are summarized below (Table 2).

**Table 2:** Categories and subcategories.

Categories	Subcategories (operational definition; qualifier)	Illustrative quote (participant code)
Increased number of roles	Giving up their own central role in their lives (caregiver’s projects/needs deprioritized as recipient’s needs become central; predominantly constraining)	“I do everything… She couldn’t survive without me by her side.” (P12, husband)
	Interpersonal synergy (episodes of attunement/cooperative coping that ease the role; ambivalent)	“I’ve had a hard time… with the fact that he had to stay at home.” (P13, wife)

Life impact	Change in status (from partner/child to “primary carer”; routines/work reconfigured; predominantly constraining)	“I’ve been at home for two years now.” (P19, daughter)
	Mustering energy at critical moments (intense bursts at clinical peaks followed by depletion; ambivalent)	“Adrenaline keeps you going… but you can burn out.” (P23, husband)
	Exhaustion and suffering (accumulated emotional/physical strain; predominantly constraining)	“Your whole world crumbles down on you…” (P13, wife)
	Clinical repercussions (palpitations, sleep disturbance, weight change, anxiety; predominantly constraining)	“Chest pain… palpitations… I’ve lost 7 kilos.” (P3, sister)

Future prospects	Hope (daily improvements sustain hope despite uncertainty; predominantly enabling)	“That’s all I hope for… that he recovers.” (P21, daughter)
	Social sensitivity (newfound appreciation for organ/blood donation; enduring gratitude and empathy towards other families; predominantly enabling)	Expressed diffusely across interviews.
	Ongoing fear and hypervigilance regarding rejection and infections; predominantly constraining	“You always live in fear of rejection.” (P13, wife)

Long‐term, ongoing needs	Meaningful information and communication with professionals (clear, compassionate, tailored across phases; ambivalent)	“Sometimes they could give more hope… it was all bad news.” (P2, wife)
	Maintaining the social and family network (presence helps; absence harms; ambivalent)	“It’s just the two of us…” (P28, wife)
	Social and financial protection (work disruption, travel/lodging costs, limited rights; predominantly constraining)	“We couldn’t come every day… guesthouse… dad quit his job.” (P19, daughter)

*Note:* Predominantly constraining = participants’ accounts mainly describe limiting or onerous effects; Ambivalent = co‐occurring costs and benefits; Predominantly enabling = accounts mainly describe strengthening or empowering effects. Qualifiers indicate interpretive orientation, not measurement. Quotes are illustrative and attributed by participant code. *Source:* Prepared by the authors.

To enhance interpretive clarity in relation to our research question, each subcategory in Table 2 is accompanied by a concise operational definition and an interpretive qualifier (predominantly constraining, ambivalent, or predominantly enabling), as warranted by the data. This qualifier is used to convey the dominant orientation of participants’ accounts for that subcategory; it is contextual and interpretive, not quantitative. A short, representative quote is provided in each row to illustrate the data–theme linkage.

### 3.1. “*I Do Everything*”: Increased Number of Roles

This category highlights the duality of the caregiving role during the LT process—where it can foster connection and teamwork in some cases but lead to significant emotional and physical strain in others.

#### 3.1.1. Giving Up Their Own Central Role in Their Lives

During the LT process, family members experience a profound transformation in their daily lives, often taking on an expanded caregiving role to meet the basic needs that the patient can no longer fulfill independently. The caregiver role becomes a central focus, frequently taking precedence over their own needs, leading them to neglect themselves in favor of supporting their relative. Thirty of the 31 participants interviewed referred to this experience, highlighting the pervasive impact of the caregiving role on their lives.

One participant, a 68‐year‐old husband caring for his wife who underwent a transplant nine months prior, expressed it poignantly:

“I do everything, everything. I’m by her side from the moment she gets up to the moment she goes to bed… Now I’ve turned into her… She couldn’t survive without me by her side.” P12, husband.

When interviewed, participants often found it difficult to talk about themselves. They displayed emotional inhibition when recounting their experiences; many redirected the conversation back to the transplant recipient, even when asked specifically about their own well‐being. Several apologized when they became emotional, as if their suffering were secondary or inappropriate. One participant remarked, “The one who’s really unwell is him, not me” (P5, wife), minimizing her own distress. The researcher often had to gently redirect the focus to the caregiver’s perspective, highlighting their tendency to prioritize the patient’s needs over their own. This reluctance to express their emotions underscores the extent to which caregiving becomes an identity of self‐suppression.

While emotional strain and exhaustion were commonly described, clear indicators of caregiver burnout were not consistently reported across participants. Some caregivers had recently assumed this role, while others had been involved in complex care for longer periods, which may explain this variability.

#### 3.1.2. Interpersonal Synergy

The adaptation to these role changes varied significantly among participants. When there was interpersonal synergy—understood here as a sense of mutual understanding, emotional attunement, and cooperative coping—caregivers described the transition into their new role as more satisfactory and manageable. This synergy fostered a sense of partnership, where both caregiver and recipient recognized and supported each other’s emotional and practical needs.

In contrast, when this synergy was lacking, caregiving often felt overwhelmingly burdensome, as if the caregiver’s efforts were invisible or unreciprocated. These differences in relational dynamics played a key role in shaping the caregiver’s emotional experience and overall resilience.

A 55‐year‐old wife of a patient who underwent a transplant 24 months prior described her struggle with this adjustment:

“…well, by the time he was on oxygen, he had to stop working… I’ve had a hard time [dealing] with the fact that he [had to stay] at home…” P13, wife.

These relational dynamics—ranging from emotional closeness to emotional distance—shaped how participants experienced their caregiving role. When mutual support was present, caregivers felt more emotionally resilient. In its absence, caregiving was often described as isolating and overwhelming.

### 3.2. “*Keep Going*” Life Impact

This category underscores the far‐reaching impact of the LT process on caregivers’ lives, highlighting the emotional, social, and physical toll they endure while supporting their loved ones.

#### 3.2.1. Change in Status

The participants reported that their relative’s illness and the LT process significantly disrupted their activities of daily living, as well as their personal, social, and work relationships. This upheaval led to a profound change in their social status and self‐perception.

A 22‐year‐old daughter of a patient who underwent an LT 16 months prior described the impact on her daily life:

“I go out some evenings, but very rarely, because I’ve also got to work the next day, at the weekends. So, [things won’t change] until [my parent] gets a bit better. I’ve been at home for two years now.” P19, daughter.

These narratives reflect how caregiving restructures one’s sense of self, often reinforcing social withdrawal. This reinforces the importance of social policies that provide support for caregivers to maintain a balanced life.

#### 3.2.2. Mustering Energy at Critical Moments

Despite these challenges, caregivers expressed a sense of satisfaction at being able to muster extraordinary energy during the most critical moments of the process, even though this was often followed by periods of intense exhaustion.

A 49‐year‐old husband of a patient who underwent an LT 6 months prior reflected on his experience:

“I mean, while he was here, it was as though the adrenaline was making you keep going, keep going, keep going. In fact, people told me ‘Be careful, ____, you’re going to burn out…’ like, ‘take care of yourself.’” P23, husband.

While resilience plays a key role, these findings highlight the risk of physical and emotional depletion. This underscores the need for caregiver self‐care strategies and long‐term coping mechanisms.

#### 3.2.3. Exhaustion and Suffering

Critical and stressful periods—such as receiving the news of the need for an LT, waiting for the transplant procedure, ICU stays, and readmissions due to complications—left participants feeling overwhelmed and out of control. These situations often resulted in significant exhaustion and suffering.

A 55‐year‐old wife of a patient 24 months post‐LT shared her feelings:

“Your whole world crumbles down on you, you know? [When you find out that] he’ll die in five years’ time if he doesn’t get a transplant.” P13, wife.

The LT process was perceived as an emotional rollercoaster, characterized by persistent suffering and a spectrum of negative emotions that contributed to physical, mental, and emotional exhaustion.

A 56‐year‐old husband of a patient who underwent an LT 28 months ago described reaching his limits:

“But there comes a time when you just can’t do it anymore. There comes a time when you run out of steam.” P8, husband.

These findings highlight the cumulative burden of caregiving, when sustained emotional distress and uncertainty lead to severe exhaustion. The chronic nature of stressors, from the initial diagnosis to the post‐transplant trajectory, creates an ongoing psychological toll. Caregivers often suppress their own emotional needs, prioritizing the well‐being of the patient, which can result in burnout.

#### 3.2.4. Clinical Repercussions

Many participants also reported a decline in their physical and emotional health, attributing these issues to the prolonged demands of caregiving during the LT process. They noted that uncertainty, particularly at critical moments, was a major source of anxiety, although this anxiety was present throughout the entire journey.

A 51‐year‐old sister of a patient 10 months post‐LT detailed the physical toll on her health:

“I get pain in my chest and heart palpitations… and a lot of blood pressure spikes… I’m being monitored by my GP… I’ve lost 7 kilos… [and that’s] after a year of all this…” P3, sister.

Caregiving extends beyond the act of providing care, influencing self‐perception, daily activities, and long‐term health. These findings highlight the emotional costs of caregiving.

### 3.3. “*Live in Fear*” Future Prospects

This category underscores the dual nature of caregivers’ future perspectives: a fragile balance between hope for the patient’s recovery and the ever‐present fear of complications and loss. It also highlights how the LT journey shaped participants’ values and heightened their awareness of the importance of donation.

The future is viewed with great uncertainty and ambivalence by caregivers. All participants expressed hope that the LT process would go well and that the patient’s health would improve. At the same time, they unanimously reported living in constant fear of complications and the looming specter of organ rejection, which remained ever‐present in their minds.

#### 3.3.1. Hope and Fear

For many participants, LT was perceived as a temporary alternative to death, offering a chance for an undefined period of life extension. While the long‐term outlook was fraught with uncertainty, they found hope in short‐term progress and small day‐to‐day improvements. All participants celebrated their relatives’ achievements, no matter how modest they were.

A 42‐year‐old daughter of a patient 28 months post‐LT shared her hopes:

“That’s all I hope for, that he gets a little better, that [the transplant] stops being rejected, that he recovers, and that’s it. That’s all we ask for.” P21, daughter.

This delicate balance between hope and fear defines the caregivers’ psychological adaptation to the post‐transplant process.

#### 3.3.2. The Specter of Rejection and Other Shadows

Despite these moments of optimism, caregivers described being haunted by the constant threat of complications and rejection of the new organ. This lingering fear created a state of vulnerability and a persistent awareness of mortality, which cast a shadow over their daily lives. Twenty‐eight of the 31 participants specifically mentioned the fear of organ rejection.

A 55‐year‐old wife of a patient 24 months post‐LT articulated this ongoing anxiety:

“You always live in fear, with the spectre of [organ] rejection. Alright, I suppose. Nothing will ever be the same [anyway].” P13, wife.

These findings illustrate how the uncertainty surrounding organ rejection extends beyond medical concerns, shaping caregivers’ emotional landscapes and reinforcing a chronic state of hypervigilance. The constant anticipation of loss prevents many caregivers from fully embracing recovery, keeping them in a state of emotional limbo.

#### 3.3.3. Social Sensitivity

Interestingly, three participants described how the experience of caring for a transplant recipient prompted them and their families to develop a newfound appreciation for organ and blood donation. Notably, this had not been a consideration prior to their involvement in the LT process.

Some participants expressed deep empathy for other families navigating similar challenges, as well as profound and ongoing gratitude toward organ donors and their families.

### 3.4. Long‐Term, Ongoing Needs

All family caregivers highlighted several ongoing needs throughout the LT process. These included receiving reliable information, maintaining open and effective communication with healthcare providers, and having access to robust family, social, and professional support networks, as well as social and financial security.

#### 3.4.1. Meaningful Information and Communication With Professionals

Some participants reported receiving insufficient information during certain phases of the process, while others felt that the information provided, particularly during the diagnostic phase, was delivered in a harsh manner. The distress caused by seeking information online also exacerbated their suffering.

A 45‐year‐old wife of a patient 13 months post‐LT reflected on the need for more hope during this phase:

“I think that sometimes, many times, they could give people more hope, don’t you think? Until he received his transplant, it was all really bad news, always giving you an ‘expiry date’, ‘you could last three years, you could last…’” P2, wife.

During the waiting phase, most participants felt that they received adequate information from the LT team. However, some admitted avoiding certain details out of fear, preferring to read only less distressing sections.

A 60‐year‐old wife of a patient 18 months post‐LT shared:

“I haven’t dared to ask about, you know, life expectancy, because I also find it extremely unsettling to ask that question in front of him.” P18, wife.

Another challenge involved the difficulty of immediately sharing the situation with other family members, as caregivers sought to protect them from emotional distress.

A 56‐year‐old husband of a patient 28 months post‐LT explained:

“If I told my family, sooner or later someone would eventually blurt it out. So, of course, to, quote unquote, ‘avoid suffering’, especially for my children’s sake, of course, I kept it from them.” P8, husband.

Despite these challenges, participants consistently expressed satisfaction and gratitude toward the LT team.

A 45‐year‐old sister of a patient 8 months post‐LT expressed her gratitude:

“We’ve been given another chance at life. I am, we are all very grateful to everyone at the Vall d’Hebron Hospital, I mean, we couldn’t be happier, they’ve given my sister her life back.” P17, sister.

#### 3.4.2. Maintaining the Social and Family Network

The presence of strong family and social support networks significantly influenced participants’ ability to cope with the process. When these networks were present, caregivers felt more supported, found it easier to delegate responsibilities, and managed their roles more effectively.

A 56‐year‐old husband of a patient 28 months post‐LT highlighted the importance of his support system:

“My sisters‐in‐law, my mother, other family members, and, above all, our neighbours helped us a lot… by keeping us company, by being with her, by helping us… Well, that, that’s crucial. I don’t know if it’s the same for everyone because I don’t think that everybody is that lucky, but we have been that lucky.” P8, husband.

In contrast, caregivers without such networks reported feeling isolated and burdened. A 57‐year‐old wife of a patient 10 months post‐LT succinctly expressed this isolation, stating: “It’s just the two of us, you know what I mean?” P28, wife.

These findings underscore the critical role of social and family networks in alleviating caregiver burden. While strong support systems facilitate emotional resilience and practical assistance, the absence of such networks intensifies feelings of loneliness, stress, and overload.

#### 3.4.3. Social and Financial Protection

Some participants emphasized the importance of the healthcare system, which provided treatment without discrimination. However, they also noted the significant financial and occupational burdens imposed by the LT process. Many caregivers reported having to make changes in their work life, such as taking sick leave, long‐term absences, or unpaid leave, and in some cases, resigning from their jobs altogether.

Additionally, the distance between participants’ homes and the hospital compounded these challenges, with many citing insufficient financial and logistical support for travel and accommodations.

A 22‐year‐old daughter of a patient 10 months post‐LT described her family’s struggles:

“There was no way we could come [to the hospital] every day. On top of the fact that my father quit his job to be able to be here and that he used to spend a lot [of money] on the guesthouse and stuff… we couldn’t come here every weekend.” P19, daughter.

This category underscores the multifaceted and ongoing needs of family caregivers, including their reliance on information, communication, and support networks, as well as the significant social and financial implications of caregiving during the LT process.

## 4. Discussion

These findings reveal the profound multidimensional impact of the LT caregiving experience. Participants described navigating emotional distress, shifts in personal and relational roles, and increased social and financial vulnerability, often prioritizing the patient’s needs above their own. While hope and resilience were evident, the burden of care remained substantial, underscoring the need for holistic interventions that address psychosocial, informational, and structural aspects of support.

This study explored the experiences, needs, and expectations of family members supporting LT recipients through the transplantation process. Guided by Meleis’ theory of transitions [18], the findings are contextualized within a framework that emphasizes adaptation and transformation during significant life changes. The theory emphasizes the interplay of personal conditions, contextual factors, and the nature of the transition, offering a structured lens to understand the complexities faced by caregivers during the LT journey. While limited studies focus specifically on LT, insights from research on other transplantation processes enrich this discussion.

Family caregivers supporting LT recipients face profound changes in their roles, adjusting familial, occupational, and social responsibilities to meet the patient’s needs. This dynamic often requires caregivers to deprioritize their own well‐being, aligning with Meleis’ assertion that role changes are central to transitional experiences [18]. Successful transitions rely on the synergy between the individual and their environment, highlighting the need for external support systems [18]. Studies by Goetzinger et al. [23] and Yagelniski et al. [24] underscore the negative correlation between caregiving overload and caregivers’ psychological well‐being, emphasizing the importance of contextual support during transitions. In our study, caregivers’ emotional inhibition was frequently observed; many minimized their own suffering or apologized when becoming emotional. Some redirected the focus back to the transplant recipient, revealing how caregiving not only consumes time and energy but also reshapes the sense of self. These identity shifts, often internalized silently, demonstrate how caregivers move into a relational role where self‐care is deprioritized. A unique finding in this study was the invigoration of energy experienced by caregivers during critical moments, potentially representing a resilience mechanism warranting further investigation.

The LT process significantly impacted caregivers’ physical and emotional health, with anxiety emerging as a predominant theme throughout the process. Feelings of fear, uncertainty, worry, anger, and guilt were commonly reported, reflecting the challenges of navigating a health‐related transition. These emotions resonate with Meleis’ framework, which describes the stressors and vulnerabilities inherent in adapting to new realities. The findings align with Ågren et al. [25], who reported elevated anxiety and depression among caregivers of transplant patients, varying across different stages of the transplantation process. Similarly, Quevedo‐Blasco et al. [26], in their systematic review on death anxiety among caregivers of chronic patients, emphasize how the anticipation of loss and prolonged caregiving responsibilities exacerbate psychological distress. Specifically, caregivers of stem cell and heart transplant patients exhibit significant rates of psychological distress, with 47% and 16% of stem cell transplant caregivers reporting clinically significant symptoms of anxiety and depression, respectively [27–29]. This highlights the critical need for targeted psychological interventions that address the unique challenges faced by caregivers during health‐related transitions.

The psychological toll observed aligns with Meleis’ focus on the conditions influencing transitions, such as situational demands and personal vulnerability. These results reinforce the importance of targeted psychological interventions to support caregivers during this demanding transition, reducing the mental health burden they experience.

The significant psychological burden experienced by caregivers in this study is consistent with evidence linking chronic stress to adverse health outcomes. McEwen [30] introduced the concept of allostatic load, which describes the cumulative physiological wear and tear resulting from chronic stress. Prolonged activation of stress response systems, such as the hypothalamic–pituitary–adrenal (HPA) axis, can lead to long‐term health consequences, including cardiovascular disease, immune dysregulation, and metabolic disorders. Similarly, Cohen et al. [31] highlighted that chronic stress not only exacerbates psychological conditions, such as anxiety and depression, but also impairs the body’s ability to regulate inflammatory processes, contributing to a range of physical illnesses.

These findings reinforce the relevance of addressing the mental health needs of caregivers, as the prolonged psychological strain observed in this study aligns with the mechanisms described by McEwen [30] and Cohen et al. [31]. Quevedo‐Blasco et al. [26] further emphasize the critical role of addressing death anxiety in caregivers, highlighting how the anticipation of loss and prolonged caregiving responsibilities exacerbate psychological distress. Interventions aimed at reducing stress and supporting caregivers’ emotional well‐being, including targeted strategies to manage death anxiety, may mitigate these negative health impacts, highlighting the importance of integrating mental health support into caregiving contexts.

Ambivalence regarding the future was another prominent theme, with caregivers expressing mixed feelings of hope and fear. While short‐term progress provided comfort, persistent vulnerability and heightened awareness of mortality created a state of emotional ambivalence. These findings align with the study by Haugh and Salyer [32], which described alternating periods of calm and tension among caregivers. Similarly, Ivarsson et al. [8] reported that fear of complications and rejection remains a significant concern among caregivers. The ongoing threat of complications and rejection perpetuated anxiety [25, 33], as noted in this study, reinforces Meleis’ emphasis on the complexity of transitions, where caregivers continually adjust to the unpredictable nature of the transplantation process [18].

Caregivers in this study highlighted the importance of reliable information, effective communication, and the availability of support networks, as well as social and financial protections. In Meleis’ framework [18], the success of a transition is influenced by the availability of resources and support systems. High‐quality information provided by healthcare professionals was seen as critical for navigating the transplant process, a finding consistent with prior studies [23–25, 34, 35].

Beyond professional information and formal networks, the quality of relational dynamics between caregivers and transplant recipients also shaped the caregiving experience. When mutual emotional and practical collaboration was present—what participants described as “working together” or “understanding each other”—caregivers reported greater emotional resilience and a shared sense of purpose. In contrast, the absence of such synergy often led to feelings of invisibility, frustration, and emotional overload. These findings align with Meleis’ emphasis on relational and environmental conditions shaping transitions [18] and suggest that interventions should address not only the caregiver’s individual needs but also the quality of caregiver–patient interactions.

Nurses are widely recognized in the nursing discipline not only as providers of clinical care but also as key facilitators of health‐related transitions, particularly in complex processes such as LT. Within the framework of Meleis’ theory of transitions [18], which guides the present study, nurses play a crucial role in supporting caregivers by offering education, emotional support, and relational guidance. By fostering trust, co‐constructing meaning, and encouraging caregivers to express their vulnerabilities and strengths, nurses facilitate a more conscious and empowered adaptation to the caregiving role. This educational and relational dimension of nursing care, grounded in an understanding of transitional needs, reflects the essence of nursing as both a science and an art. Integrating this approach into nursing practice can enhance caregivers’ resilience and contribute to more humanized, responsive care systems [36].

Interestingly, while Ivarsson et al. [35] and Moloney et al. [37] noted a gap between the information relatives received and the information they needed, this was not observed in our study, as participants generally felt well‐informed during the LT process. This finding could indicate the presence of facilitating conditions that eased the transition for caregivers, as emphasized in Meleis’ theory.

The presence of robust support networks significantly influenced caregivers’ ability to cope. Participants who had access to family or social support reported feeling more capable of managing their caregiving roles, reflecting Meleis’ view that environmental and relational factors are essential for successful transitions, and a finding consistent with previous research [8, 32, 34, 38–40].

Conversely, financial and logistical challenges posed significant barriers for many caregivers, particularly for those living far from the hospital. These findings align with studies reporting financial strain and insufficient social support for caregivers during transplantation [23, 25, 34, 40].

### 4.1. Study Strengths and Limitations

The single‐center design and the pandemic‐related suspension of nonessential research—which extended the overall timeline—may affect transferability; COVID‐19 itself was not an object of inquiry and is mentioned only to contextualize data‐collection logistics. However, the robust integration of results with insights from international literature allows for meaningful extrapolation to comparable contexts. Credibility is further supported by the application of comprehensive qualitative methodologies and a well‐rounded literature review, which situates the study within a broader research framework. The involvement of a multidisciplinary team enriched the process by incorporating varied expertise, enhancing both the study’s design and the interpretation of its findings. Adherence to rigorous ethical standards added an additional layer of reliability.

### 4.2. Implications for Nursing Practice

Enhancing the caregiver experience in LT is essential for patient recovery and quality care. Guided by Meleis’ theory of transitions, nurses can develop personalized care plans to address caregivers’ needs, such as education on medication management, post‐transplant lifestyle adjustments, and strategies to manage emotional strain. Regular follow‐ups, including telehealth options, can monitor caregiver well‐being and provide timely support.

Continuous education for nursing professionals is crucial to improve caregiver support. Training on caregiver burden, resilience‐building, and effective communication equips nurses to deliver tailored interventions that reduce stress and enhance well‐being. Further research is needed to develop and validate specific interventions for caregivers in LT, reinforcing the vital role of nurses throughout the process.

This study lends a voice to the family members who accompany LT recipients throughout the transplantation process, highlighting their critical role in patient care.

## 5. Conclusions

The findings emphasize the need to address caregivers’ challenges, including financial strain, work–life balance, and emotional well‐being, which require action from healthcare systems and relevant authorities. The results also underscore the vital role of nurses in providing guidance and support, advocating for caregiver‐centered interventions to improve care quality.

Future efforts should focus on developing holistic strategies that enhance the support and resilience of caregivers, ensuring their needs are met during this complex transition.

## Conflicts of Interest

This research is part of the lead investigator’s doctoral thesis within the doctoral program in Medicine and Translational Research at the University of Barcelona. The other authors declare no conflicts of interest.

## Funding

No funding was received for this research.

## Data Availability

Data are available upon request due to privacy/ethical restrictions. The data that support the findings of this study are available upon request from the corresponding author. The data are not publicly available due to privacy or ethical restrictions.

## References

[1] Román A. , Ussetti P. , Solé A. et al., Normativa Para La Selección De Pacientes Candidatos a Trasplante Pulmonar, Archivos de Bronconeumología. (2011) 47, no. 6, 303–309, 10.1016/j.arbres.2011.03.007. 21536362

[2] Bos S. , Vos R. , Van Raemdonck D. , and Verleden G. , Survival in Adult Lung Transplantation: Where Are we in 2020?, Current Opinion in Organ Transplantation. (2020) 25, no. 3, 268–273, 10.1097/MOT.0000000000000753. 32332197

[3] Organización Nacional de Trasplantes , Memoria De Actividad De Donación Y Trasplante. España 2023. Organización Nacional De Trasplantes, 2024, https://www.ont.es/wp-content/uploads/2024/03/actividad-de-donacion-y-trasplante-espana-2023.pdf.

[4] Chan E. , Hayanga J. , Tuft M. , Morrell M. , and Sanchez P. , Access to Lung Transplantation in the United States: The Potential Impact of Access to a High-Volume Center, Transplantation. (2020) 104, no. 7, e199–e207, 10.1097/TP.0000000000003259. 32569004 PMC7885168

[5] Solé A. , Pérez I. , Vázquez I. et al., Patient-Reported Symptoms and Functioning as Indicators of Mortality in Advanced Cystic Fibrosis: A New Tool for Referral and Selection for Lung Transplantation, The Journal of Heart and Lung Transplantation. (2016) 35, no. 6, 789–794, 10.1016/j.healun.2016.01.1223, 2-s2.0-84962162246. 27021279

[6] Shahabeddin Parizi A. , Krabbe P. F. M. , Verschuuren E. A. M. et al., Patient-Reported Health Outcomes in Long-Term Lung Transplantation Survivors: A Prospective Cohort Study, American Journal of Transplantation. (2018) 18, no. 3, 684–695, 10.1111/ajt.14492, 2-s2.0-85031325489. 28889654 PMC5836864

[7] Mollberg N. , Farjah F. , Howell E. , Ortiz J. , Backhus L. , and Mulligan M. , Impact of Primary Caregivers on Long-Term Outcomes After Lung Transplantation, The Journal of Heart and Lung Transplantation. (2014) 34, no. 1, 59–64, 10.1016/j.healun.2014.09.022, 2-s2.0-84919958420. 25447578

[8] Ivarsson B. , Ekmehag B. , and Sjöberg T. , Relatives’ Experiences Before and After a Heart or Lung Transplantation, Heart & Lung. (2014) 43, no. 3, 198–203, https://www.ncbi.nlm.nih.gov/pubmed/24680630, 10.1016/j.hrtlng.2014.02.005, 2-s2.0-84899937377. 24680630

[9] Goetzmann L. , Moser K. S. , Vetsch E. et al., What do Patients Think After a Lung Transplantation About Their Self, Lung, and Social Network? A Quantitative Analysis of Categorical Interview Data, GMS Psycho-Social-Medicine. (2006) 3. PMC2736508 19742273

[10] Meltzer L. and Rodrigue J. , Psychological Distress in Caregivers of Liver and Lung Transplant Candidates, Journal of Clinical Psychology in Medical Settings. (2001) 8, no. 3, 173–180, 10.1023/A:1011317603415, 2-s2.0-0034854496.

[11] George E. , Large A. , Boujoukos A. , and Tuttle R. , Respiratory Care of the Postoperative Lung Transplantation Patient, Critical Care Nursing Quarterly. (1996) 19, no. 3, 59–69, 10.1097/00002727-199619030-00006, 2-s2.0-0029909009. 8981852

[12] Glaze J. , Brooten D. , Youngblut J. , Hannan J. , and Page T. , The Lived Experiences of Caregivers of Lung Transplant Recipients, Progress in Transplantation. (2021) 31, no. 4, 299–304, 10.1177/15269248211046034. 34704858

[13] Yorke J. and Cameron-Traub E. , Patients’ Perceived Care Needs Whilst Waiting for a Heart or Lung Transplant, Journal of Clinical Nursing. (2008) 17, no. 5A, 78–87, 10.1111/j.1365-2702.2007.02078.x, 2-s2.0-65249181151. 18298758

[14] Adegunsoye A. , Strek M. , Garrity E. , Guzy R. , and Bag R. , Comprehensive Care of the Lung Transplant Patient, Chest. (2017) 152, no. 1, 150–164, 10.1016/j.chest.2016.10.001, 2-s2.0-85018258572. 27729262 PMC6026268

[15] Suzuki H. and Yoshino I. , A Narrative Review on the Management of Patients Awaiting Lung Transplantation in Japan, Journal of Thoracic Disease. (2023) 15, no. 10, 5856–5862, 10.21037/jtd-22-1690. 37969266 PMC10636442

[16] El Hadi S. N. , Zanotti R. , and Danielis M. , Lived Experiences of Persons With Heart Transplantation: A Systematic Literature Review and Meta-Synthesis, Heart & Lung, 10.1016/j.hrtlng.2024.10.013. 39481147

[17] Al‐Sheikh Hassan M. , The Use of Husserl’s Phenomenology in Nursing Research: A Discussion Paper, Journal of Advanced Nursing. (2023) 79, no. 8, 3160–3169, 10.1111/jan.15564. 36718849

[18] Meleis A. I. , Transitions Theory: Middle-Range and Situation-Specific Theories in Nursing Research and Practice, 2010, Springer Publishing Company.

[19] Colaizzi P. F. , Valle R. S. and King M. , Psychological Research as the Phenomenologist Views it, Existential-Phenomenological Alternatives for Psychology, 1978, Oxford University Press, 48–71.

[20] O’Brien B. C. , Harris I. B. , Beckman T. J. , Reed D. A. , and Cook D. A. , Standards for Reporting Qualitative Research: A Synthesis of Recommendations, Academic Medicine. (2014) 89, no. 9, 1245–1251, 10.1097/ACM.0000000000000388, 2-s2.0-84908022244. 24979285

[21] Denzin N. K. and Lincoln Y. S. , Denzin N. K. and Lincoln Y. S. , Introduction: The Discipline and Practice of Qualitative Research, The Sage Handbook of Qualitative Research, 2005, 3rd edition, Sage, 1–32.

[22] Lincoln Y. S. and Guba E. G. , Naturalistic Inquiry, 1985, Sage Publications.

[23] Goetzinger A. M. , Blumenthal J. A. , O’Hayer C. V. et al., Stress and Coping in Caregivers of Patients Awaiting Solid Organ Transplantation, Clinical Transplantation. (2012) 26, no. 1, 97–104, 10.1111/j.1399-0012.2011.01431.x, 2-s2.0-84856625938. 21395692 PMC3635131

[24] Yagelniski A. , Rosaasen N. , Cardinal L. , Fenton M. , Tam J. , and Mansell H. , A Qualitative Study to Explore the Needs of Lung Transplant Caregivers, Progress in Transplantation. (2020) 30, no. 3, 243–248, 10.1177/1526924820933842. 32552359

[25] Ågren S. , Sjöberg T. , Ekmehag B. , Wiborg M. B. , and Ivarsson B. , Psychosocial Aspects Before and up to 2 Years After Heart or Lung Transplantation: Experience of Patients and Their Next of Kin, Clinical Transplantation. (2017) 31, no. 3, 10.1111/ctr.12905, 2-s2.0-85012050815. 28039882

[26] Quevedo-Blasco R. , Díaz-Román A. , and Vega-García A. , Death Anxiety in Caregivers of Chronic Patients, Healthcare. (2024) 12, no. 1, 10.3390/healthcare12010107. PMC10871074 38201013

[27] Vovlianou S. , Koutlas V. , Papoulidou F. et al., #5602 Depression and Anxiety in Family Caregivers of End Stage Kidney Disease Patients in Greece: A Comparison Between Dialysis and Kidney Transplantation, Nephrology Dialysis Transplantation. (2023) 38, no. Supplement_1, 10.1093/ndt/gfad063c_5602.

[28] Topping C. , Nelson A. , Jacobs J. , Greer J. , Temel J. , and El-Jawahri A. , Relationship Between Caregiver Burden and Psychological Distress Among Stem Cell Transplant (SCT) Recipients Prior to Transplant, Journal of Clinical Oncology. (2020) 38, no. 15_suppl, 10.1200/jco.2020.38.15_suppl.12120.

[29] Waldman L. , Nelson A. , Jacobs J. et al., Anxiety and Depression Symptoms in Caregivers Prior to Hematopoietic Stem Cell Transplantation (HCT), Transplantation and Cellular Therapy. (2021) 27, no. 6, 517.e1–517.e5, 10.1016/j.jtct.2021.03.002. PMC8217210 33812804

[30] Mcewen B. S. , Protective and Damaging Effects of Stress Mediators, New England Journal of Medicine. (1998) 338, no. 3, 171–179, 10.1056/NEJM199801153380307, 2-s2.0-0032518022. 9428819

[31] Cohen S. , Janicki-Deverts D. , and Miller G. E. , Psychological Stress and Disease, Jama. (2007) 298, no. 14, 1685–1687, 10.1001/jama.298.14.1685, 2-s2.0-35348831738. 17925521

[32] Haugh K. H. and Salyer J. , Needs of Patients and Families During the Wait for a Donor Heart, Heart & Lung. (2007) 36, no. 5, 319–329, 10.1016/j.hrtlng.2006.11.003, 2-s2.0-34548407735. 17845878

[33] Ivarsson B. , Ingemansson R. , and Sjöberg T. , Experiences of Supportive Care When Waiting for a Lung re-Transplantation, Sage Open Medicine. (2017) 5, 10.1177/2050312117697151. PMC5433791 28540044

[34] Sadala M. L. A. , Stolf N. G. , Bocchi E. A. , and Bicudo M. A. V. , Caring for Heart Transplant Recipients: The Lived Experience of Primary Caregivers, Heart & Lung: The Journal of Acute and Critical Care. (2013) 42, no. 2, 120–125, 10.1016/j.hrtlng.2012.09.006, 2-s2.0-84875248440. 23083537

[35] Ivarsson B. , Ekmehag B. , and Sjöberg T. , Waiting for a Heart or Lung Transplant: Relatives’ Experience of Information and Support, Intensive and Critical Care Nursing. (2014) 30, no. 4, 188–195, https://www.ncbi.nlm.nih.gov/pubmed/24742688, 10.1016/j.iccn.2014.03.003, 2-s2.0-84901677206. 24742688

[36] Bailey D. E. , Steinhauser K. E. , Hendrix C. C. , and Tulsky J. A. , Nurses’ Roles in Facilitating Transitions: A Qualitative Study of Patients and Caregivers Coping With Advanced Chronic Illness, Journal of Clinical Nursing. (2014) 23, no. 5–6, 735–745, 10.1016/j.pec.2020.03.012.

[37] Moloney S. , Cicutto L. , Hutcheon M. , and Singer L. , Deciding About Lung Transplantation: Informational Needs of Patients and Support Persons, Progress in Transplantation. (2007) 17, no. 3, 183–192, 10.1177/152692480701700305. 17944157

[38] Kurz J. M. C. , Vulnerability of Well Spouses Involved in Lung Transplantation, Journal of Family Nursing. (2002) 8, no. 4, 353–370, 10.1177/107484002237512, 2-s2.0-14844335044.

[39] Lannon H. , Code J. , Poole J. , Simpson C. , and Badh V. , Patients and Caregivers Perspectives of the Connection Between Home and the Transplant Journey, Heart & Lung: Journal of Critical Care. (2023) 57, 265–270, 10.1016/j.hrtlng.2022.10.008. 36332350

[40] Pawlow P. , Blumenthal N. , Christie J. , Matura L. , Aryal S. , and Ersek M. , The Supportive Care Needs of Primary Caregivers of Lung Transplant Candidates, Journal of Pain and Symptom Management. (2021) 62, no. 5, 918–926, 10.1016/j.jpainsymman.2021.05.004. 33992758

